# New adaptive statistical iterative reconstruction ASiR‐V: Assessment of noise performance in comparison to ASiR

**DOI:** 10.1002/acm2.12253

**Published:** 2018-01-24

**Authors:** Paolo De Marco, Daniela Origgi

**Affiliations:** ^1^ Medical Physics Unit European Institute of Oncology Milan Italy

**Keywords:** computed tomography, iterative reconstruction, noise power spectrum

## Abstract

**Purpose:**

To assess the noise characteristics of the new adaptive statistical iterative reconstruction (ASiR‐V) in comparison to ASiR.

**Methods:**

A water phantom was acquired with common clinical scanning parameters, at five different levels of CTDI_vol_. Images were reconstructed with different kernels (STD, SOFT, and BONE), different IR levels (40%, 60%, and 100%) and different slice thickness (ST) (0.625 and 2.5 mm), both for ASiR‐V and ASiR. Noise properties were investigated and noise power spectrum (NPS) was evaluated.

**Results:**

ASiR‐V significantly reduced noise relative to FBP: noise reduction was in the range 23%–60% for a 0.625 mm ST and 12%–64% for the 2.5 mm ST. Above 2 mGy, noise reduction for ASiR‐V had no dependence on dose. Noise reduction for ASIR‐V has dependence on ST, being greater for STD and SOFT kernels at 2.5 mm. For the STD kernel ASiR‐V has greater noise reduction for both ST, if compared to ASiR. For the SOFT kernel, results varies according to dose and ST, while for BONE kernel ASIR‐V shows less noise reduction. NPS for CT Revolution has dose dependent behavior at lower doses. NPS for ASIR‐V and ASiR is similar, showing a shift toward lower frequencies as the IR level increases for STD and SOFT kernels. The NPS is different between ASiR‐V and ASIR with BONE kernel. NPS for ASiR‐V appears to be ST dependent, having a shift toward lower frequencies for 2.5 mm ST.

**Conclusions:**

ASiR‐V showed greater noise reduction than ASiR for STD and SOFT kernels, while keeping the same NPS. For the BONE kernel, ASiR‐V presents a completely different behavior, with less noise reduction and modified NPS. Noise properties of the ASiR‐V are dependent on reconstruction slice thickness. The noise properties of ASiR‐V suggest the need for further measurements and efforts to establish new CT protocols to optimize clinical imaging.

## 1 FULL PAPER

In the U.S.A., more than 60 million examinations were performed in 2006^1^ and the number of exams in the past decade has continued to increase. Along with the number of CT examinations, the radiation exposure and related concerns about its adverse effects have also grown.^2^, ^3^, ^4^ Despite being no scientific evidence about the carcinogenic effect of ionizing radiations at low doses,^5^, ^6^ the ALARA (“As low as reasonably achievable”) principle recommends the optimization of the CT examinations.

Correspondingly, different acquisition strategies such as tube current modulation, both angular (i.e., in the x‐y plane) and longitudinal (i.e., along the z‐axis), and kV modulation according to patient size have been developed and successfully implemented on modern scanners for the purpose of dose reduction. More recently, iterative reconstruction (IR) algorithms implemented on CT scanner by manufacturers have emerged as a very promising dose reduction strategy.^7^

Currently, filtered back‐projection (FBP) is the most used reconstruction algorithm in CT. FBP relies on several assumptions, which are not exactly fulfilled in practice. While FBP is an adequate reconstruction method in most situation, when lower doses are used, the higher noise in the projections leads to noisy images with streak artifacts and poor low‐contrast detectability.

Iterative reconstruction algorithms have the capability to overcome the FBP limitations, thus allowing the formation of images with good quality at lower doses compared to FBP.^8^, ^9^, ^10^, ^11^

CT vendors have developed their own IR algorithms, in particular GE introduced at first ASiR, an algorithm working in the raw‐data domain to model the noise and the object.^12^ ASiR was followed by VEO, a full model‐based IR algorithm that implemented the modeling of the entire system, including physics and optics modeling.^13^, ^14^

The adaptive statistical iterative reconstruction (ASiR‐V) is a recently released IR algorithm developed by GE. With characteristics between those of ASiR and VEO, ASiR‐V presents a more advanced noise and object modeling than ASiR, and it has implemented physics modeling. The most time‐consuming part of iterative reconstruction, the system optics modeling, is de‐emphasized in ASiR‐V, leading to fast reconstruction times, comparable to those of FBP and ASiR.^15^, ^16^

The aim of this work is to assess the noise characteristics of the new ASiR‐V in comparison with the ASiR.

## 2 MATERIALS AND METHODS

Measurements were performed on two GE CT scanners: a CT Discovery 750 HD (software version gmp_hde.74), equipped with the ASiR and a CT Revolution (software version revo_1.5_m3a.46), equipped with the new ASiR‐V. A GE water phantom (30 cm long) was acquired in helical mode with the following scanning parameters: 120 kVp, “Large Body” scanning field of view (SFOV = 500 mm), 0.5 s rotation time, pitch close to 1 (0.984 for both scanners) and 40 mm (0.625 mm × 64) total collimation. Scans covered the whole length of the phantom.

Tube current without modulation was set to yield a CTDI_vol_ as close as possible to 1, 2, 4, 7, and 15 mGy, according to the characteristics of the scanners (Table 1).

**Table 1:** Nominal and actual CTDI_vol_ for the acquisitions on the two scanners

Nominal CTDI_vol_ (mGy)	CT Discovery 750HD	CT Revolution
	Actual CTDI_vol_ (mGy)	Actual CTDI_vol_ (mGy)
1	0.95	1.04
2	1.91	1.90
4	3.98	3.97
7	7.00	6.91
15	14.93	15.02

Three different kernels were used for the FBP reconstructions: standard (labeled as STD), SOFT and BONE. Images were also reconstructed with 3 different levels of ASiR and ASiR‐V (40%, 60%, and 100%) at slice thicknesses (ST) of 0.625 and 2.5 mm. The display field of view (DFOV) of all reconstructed images was 250 mm.

Before all the acquisitions, both scanners were successfully tested according to the respective current quality assurance protocols, with the measured CTDI_vol_ being within ±10% of the value displayed by the console.

Noise reduction relative to FBP as a function of dose, kernel and slice thickness was investigated and noise properties in the frequency domain were evaluated with the noise power spectrum (NPS), as described in the following paragraphs.

### 2.A Noise assessment

Noise was evaluated as the standard deviation (SD) of the Hounsfield units (HU) in a square ROI of 224 × 224 pixels centered in the axial images of the water phantom using ImageJ.^17^

The SDs were evaluated on 120 and 30 images for the slice thicknesses of 0.625 and 2.5 mm, respectively, to cover the same length of 75 mm across the phantom.

The SD of CT number was plotted versus the CTDI_vol_^−0.5^ in order to assess the power‐law behavior of noise in the reconstructed images.

Noise reduction in the iterative reconstruction relative to the FBP was evaluated for all the iterative levels and for all kernels as

(1) $$Noise\;\;reduction\;\;{\left(\%\right)}_{i}=\frac{S{D^{\mathrm{FBP}}}_{i}-S{D^{\mathrm{IR}}}_{i}}{S{D^{\mathrm{FBP}}}_{i}}\times 100$$

where the subscript i, referring to the slice, ranges from 1 to 120 or 1 to 30, depending on slice thickness. Noise reduction was then averaged over the i slices.

Statistical significance of noise reduction in the iterative reconstruction strategies relative to FBP was evaluated with a two‐sample t‐test.

To assess the statistical significance of noise reduction relative to FBP of ASiR‐V in comparison to ASiR, a two‐sample t‐test was performed.

In both cases, a *P*‐value of 0.05 has been chosen to reject the null hypothesis of no differences in noise reduction.

### 2.B Noise power spectrum

The above assessment of SD of HU values is not enough to fully assess the noise characteristics associated with a reconstruction process. For this, it is important to evaluate the frequency distribution of the noise, as described by the noise power spectrum (NPS). The magnitude of the NPS reflects the degree of randomness at every spatial frequency and the integral of the NPS yields the variance.^18^ NPS is defined as the Fourier transform of the autocovariance function, but it is commonly determined by taking the modulus of the 2‐D discrete Fourier transform FT_d_ of the intensity of the noise‐only image.^19^

(2) $$NPS_{d}\left(f_{x},f_{y}\right)=\frac{\Delta x\Delta y}{N_{x}N_{y}}{\left|FT_{d}\left[N\left(x,y\right)\right]\right|}^{2}$$

where ∆_k_ denotes the pixel size and N_k_ the number of pixels of the noise‐only image N(x,y), along the k axis.

The noise‐only images were obtained by subtracting 2 consecutive scans S_1_ and S_2_ and dividing by a factor of $\sqrt{2}$ to account for the doubling of variance in the images after the subtraction.

(3) $$N\left(x,y\right)=\frac{1}{\sqrt{2}}\left(S_{1}-S_{2}\right)$$

Noise power spectra were calculated using a macro of ImageJ.

**Figure 1:**
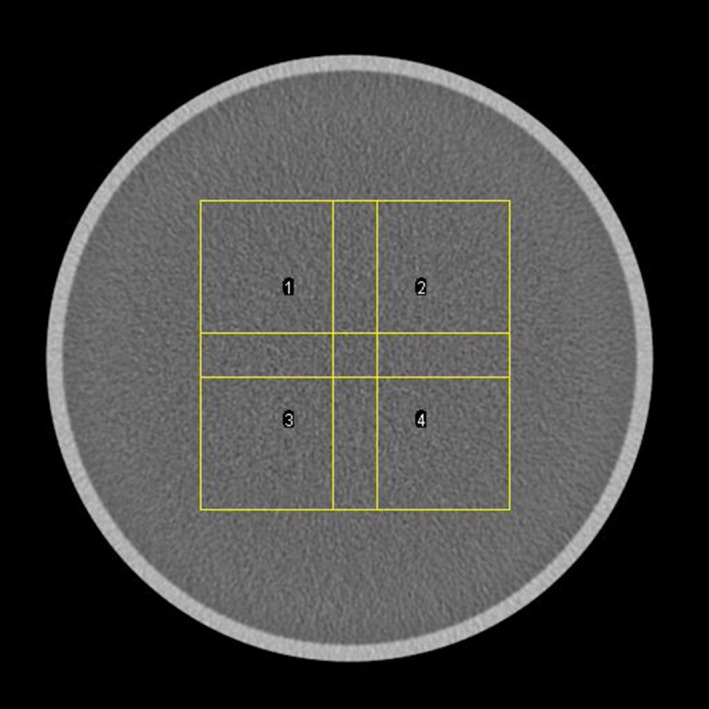
Scheme of the four ROIs used for the NPS evaluation.

For the calculations, 4 ROIs of 128 × 128 pixels, each overlapping to its neighbor by 32 pixels in the horizontal and vertical directions, were extracted (see Fig. 1). This image was then zero‐padded to a 512 × 512 image.

The Fourier transform was then calculated and the square of the magnitude was taken. Calculation was performed on 80 and 20 images (for slice thicknesses of 0.625 and 2.5 mm, respectively, in order to cover the same length of 50 mm across the phantom) and NPS_d_(f_x_, f_y_) was averaged over the 320 and 80 ROIs. Taking into account that, when calculated at the phantom isocenter, NPS_d_ (f_x_, f_y_) has a rotational symmetry, a 1D NPS(f_xy_) curve was obtained by averaging the NPS(f_x_, f_y_) values corresponding to the same radial frequency.

Each spectrum, was quantified by its peak frequency (f_peak_) and its mean frequency (f_mean_), defined as

(4) $$f_{\mathrm{mean}}=\frac{\int \int f_{\mathrm{xy}}NPS\left(f_{\mathrm{xy}}\right)df_{\mathrm{xy}}}{\int \int NPS\left(f_{\mathrm{xy}}\right)df_{\mathrm{xy}}}$$

## 3 RESULTS

### 3.A NOISE ASSESSMENT

**Figure 2:**
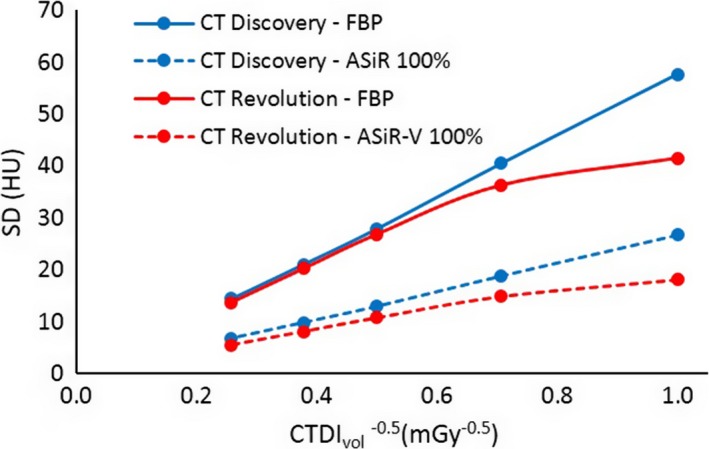
Standard Deviation (SD) as a function of CTDI_vol_.

For the FBP of the CT Discovery, we found the expected relationship between SD and CTDI_vol_ (σ ∝ CTDI_vol_^−0.5^) (Fig. 2).

On the other hand, for the CT Revolution, in the evaluation of SD as a function of CTDI_vol_, we observed that for lower doses (1 and 2 mGy), noise in the FBP varies from the well‐known power law. This scanner‐specific behavior was also present in the noise for ASiR‐V and was true for all the three kernels for both slice thicknesses. A possible explanation for this behavior is that the CT Revolution may apply a low‐pass filter to its reconstruction.

ASiR‐V significantly reduced noise relative to FBP for all dose levels and for all kernels: noise reduction was in the range 23%–60% for a 0.625 mm slice thickness and 12%–64% for the 2.5 mm slice thickness.

Noise reduction for ASiR‐V had no dependence on dose, above a CTDI_vol_ of 2 mGy.

**Figure 3:**
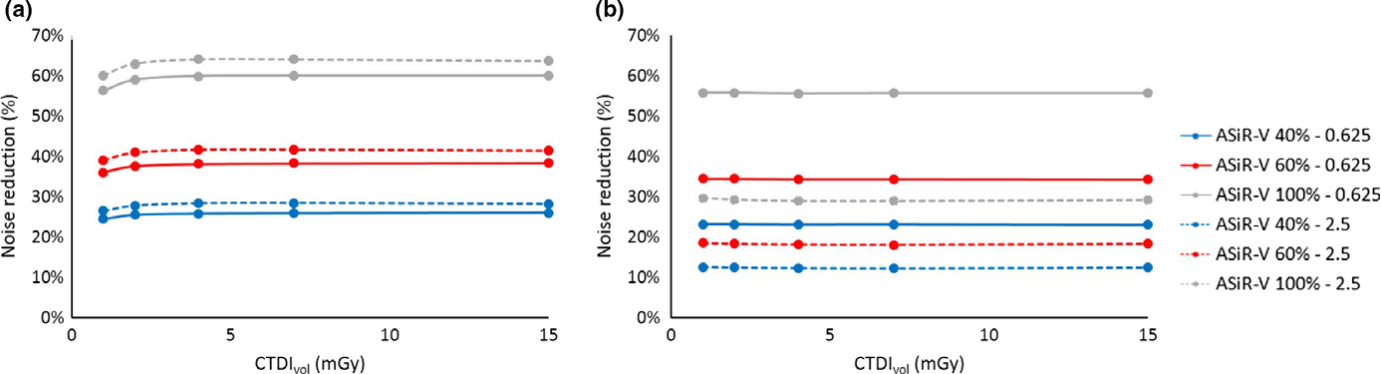
Noise reduction for the ASiR‐V as a function of slice thickness (0.625 mm solid lines −2.5 mm dashed lines) for kernel STD (a) and BONE (b).

Slice thickness had impact on noise reduction: in particular, for STD [Fig. 3(a)] and SOFT kernels, noise reduction is greater at 2.5 mm, while for BONE kernel the behavior is reversed [Fig. 3(b)]

The percentage noise reduction in ASiR‐V relative to FBP for the noise‐independent dose‐range (i.e., above 4 mGy) are presented in Table 2.

**Table 2:** Percentage noise reduction for ASiR‐V relative to FBP for slice thickness 0.625/2.5 mm

Strength of ASiR‐V	Kernel STD	Kernel SOFT	Kernel BONE
40%	26/28	23/26	23/12
60%	38/41	34/38	34/18
100%	60/64	53/59	56/29

**Figure 4:**
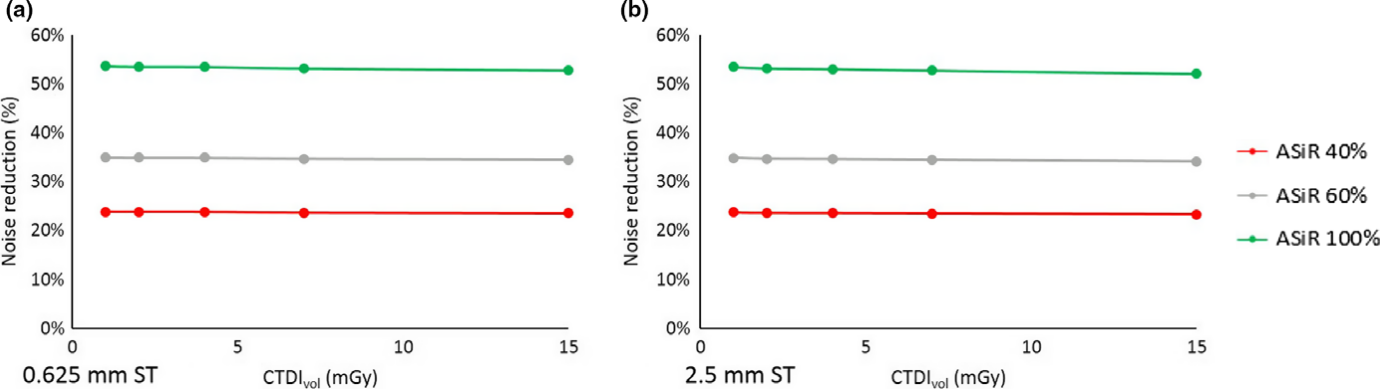
Noise reduction for the ASiR with the STD kernel with 0.625 mm (a) and 2.5 mm (b) slice thickness.

ASiR had no dependence on dose or slice thickness for all three kernels, as illustrated in Fig. 4 for the STD kernel.

If we compare the noise reduction relative to FBP, for all the reconstruction configurations (dose‐IR level‐slice thickness) with STD kernel, ASiR‐V has greater noise reduction than ASiR. (*P* < 0.01).

The results differ for the other kernels, however, due to the peculiar behavior of the ASiR‐V. For the SOFT kernel and slice thickness 0.625 mm, ASiR‐V shows an increase in noise reduction above 4 mGy (*P* < 0.01), for 2 mGy the noise reduction is roughly the same, while for 1 mGy we observe ASiR had the greater impact. If we consider a slice thickness of 2.5 mm, ASiR‐V reduced noise more than ASiR at every level of IR and at every dose (*P* < 0.01).

**Figure 5:**
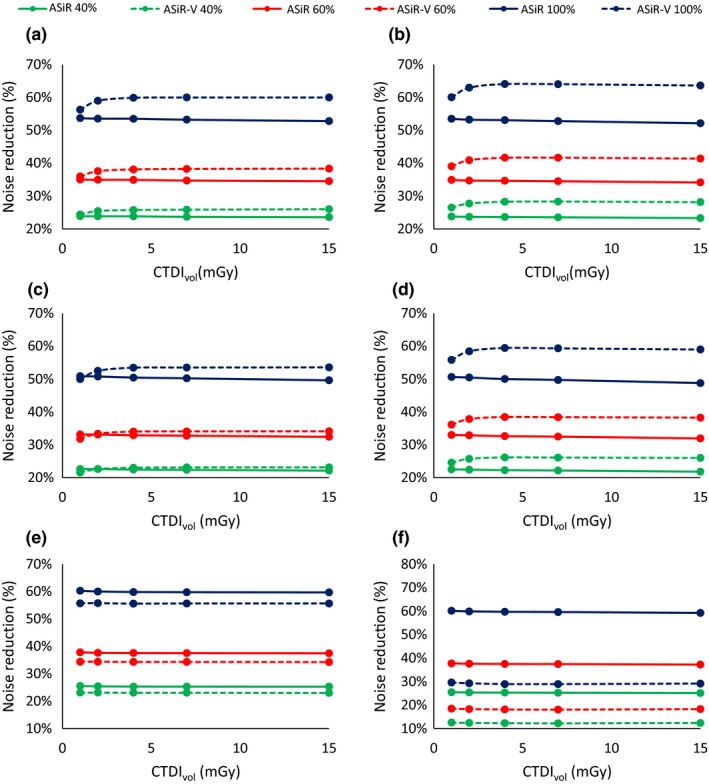
Comparison of noise reduction between ASiR and ASiR‐V with different kernels and different slice thickness: STD 0.625 mm (a) and 2.5 mm (b); SOFT 0.625 mm (c) and 2.5 mm (d); BONE 0.625 mm (e) and 2.5 mm (f).

For the BONE kernel the situation is reversed, with ASiR‐V showing less noise reduction at 0.625 mm, then the peculiar behavior at 2.5 mm reinforce this trend, as the impact in noise reduction is far lower in comparison to ASiR (*P* < 0.01). These results are shown in Fig. 5.

Complete results for noise reduction are provided in Tables A1, A2, A3, A4, A5, A6.

## 4 NOISE POWER SPECTRUM

### 4.A CT Revolution and ASiR‐V

**Figure 6:**
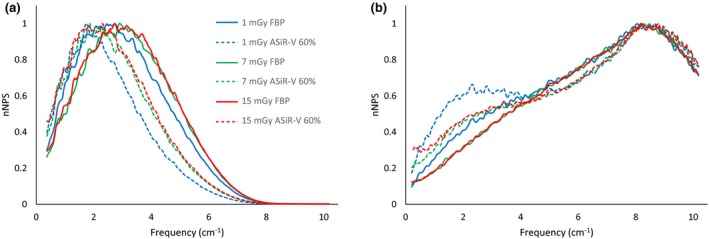
Dose‐dependent behavior below 4 mGy for the CT Revolution Normalized NPS (nNPS) are shown for kernel STD (a) and BONE (b).

The noise power spectrum of the CT Revolution for FBP at lower doses (i.e., 1 and 2 mGy) presents a shift toward lower frequencies for the STD and SOFT kernels, and a different behavior for BONE kernel (Fig. 6).

At doses above 4 mGy, the FBP spectra of the CT Revolution are almost identical, losing the dose‐dependent shift. The left shift of the FBP spectra at lower doses is reflected in the NPS of the iterative reconstructions, for all levels of IR and for all kernels.

For the STD and SOFT kernels, with fixed dose, we observe a shift toward lower frequencies when images are reconstructed using the iterative algorithm instead of the FBP. The shift is greater for higher levels (i.e., higher percentages) of the ASiR‐V.

For the kernel BONE, on the other hand, as the level of ASiR‐V increases, there is negligible shift of the high‐frequency peak and a second low‐frequency peak becomes more evident at increasing ASiR‐V percentage.

**Figure 7:**
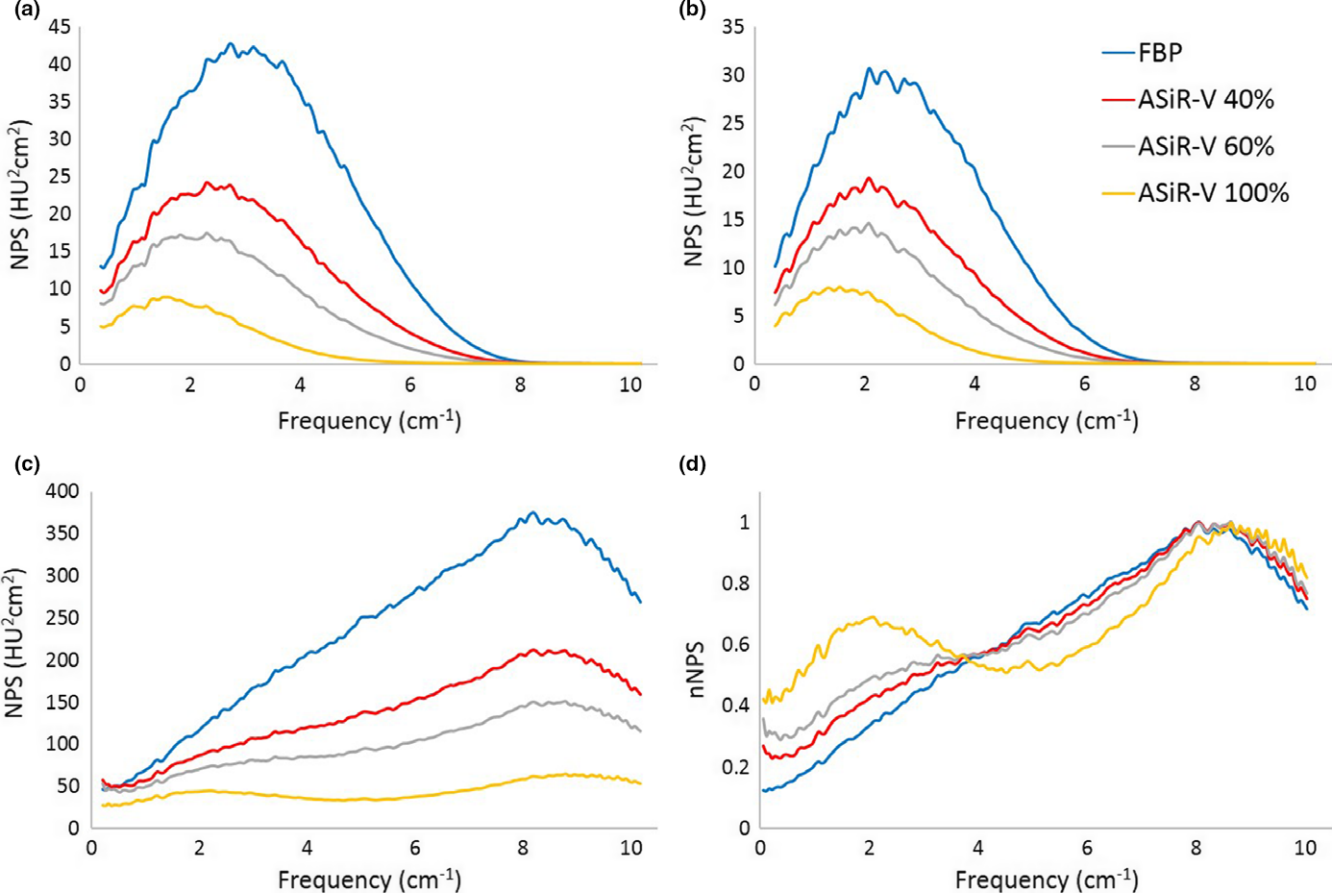
Noise Power Spectra of the ASiR‐V with different kernels at increasing IR level: kernel STD (a), SOFT (b), BONE (c), BONE normalized to the maximum (d).

Figure 7 summarizes all these results.

### 4.B ASiR‐V versus ASiR

NPS for ASiR‐V were then compared to the spectra obtained with ASiR, for the three kernels, calculated at 0.625 mm.

At first, differences in the FBPs of the two scanner have been taken into account.

**Figure 8:**
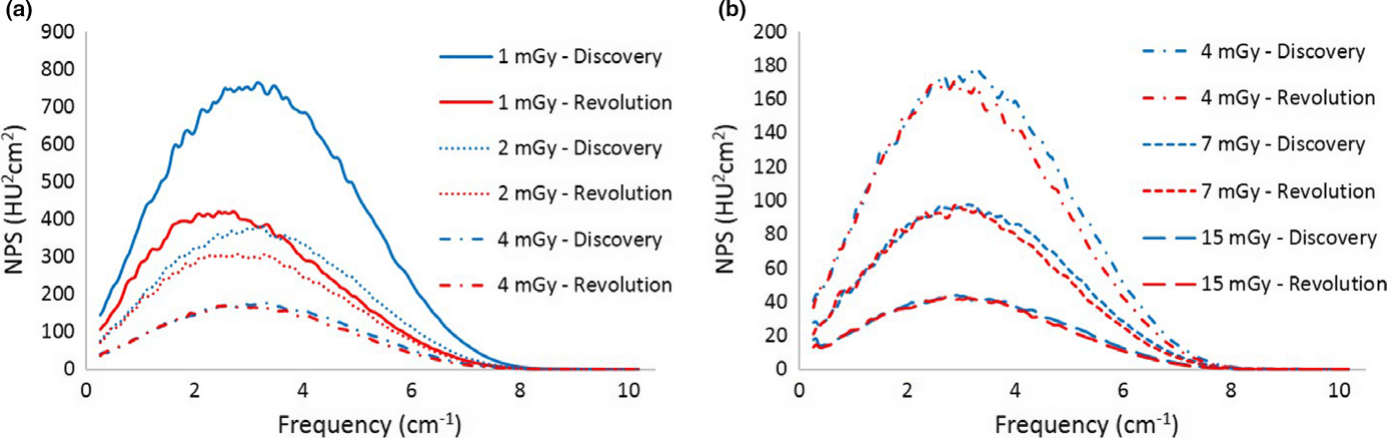
FBP spectra with STD kernel for CT Discovery and CT Revolution at different dose levels: 1–4 mGy (a), 4–15 mGy (b).

**Figure 9:**
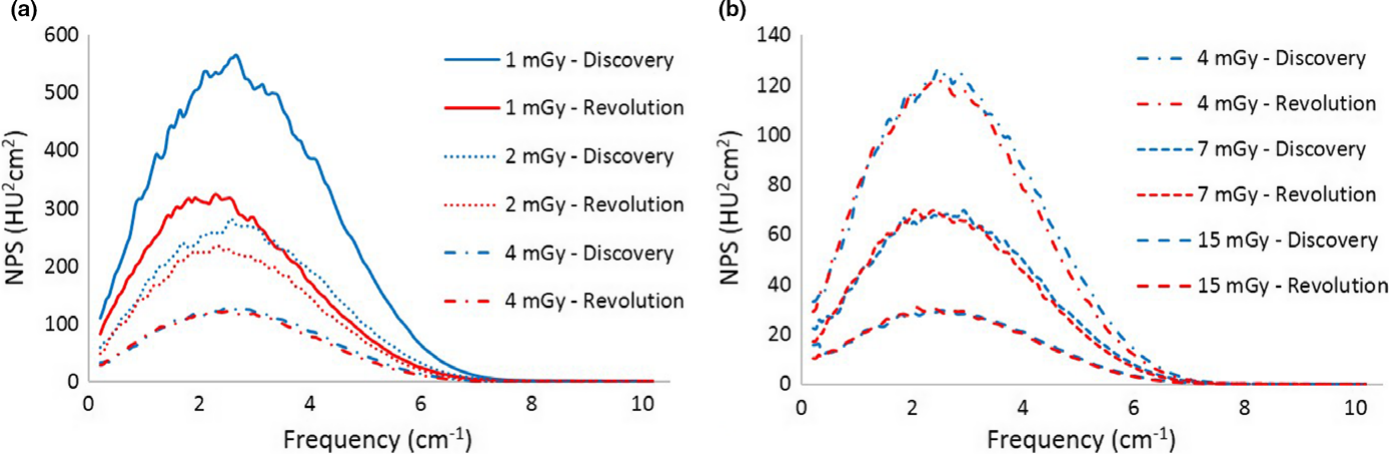
FBP spectra with SOFT kernel for CT Discovery and CT Revolution at different dose levels:1–4 mGy (a), 4–15 mGy (b).

**Figure 10:**
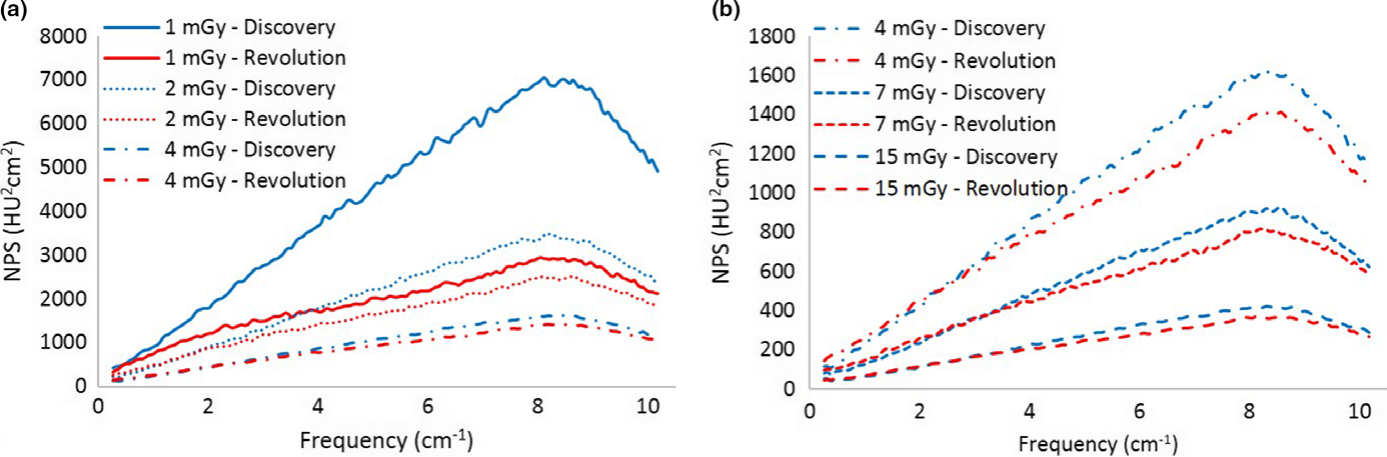
FBP spectra with BONE kernel for CT Discovery and CT Revolution at different dose levels: 1–4 mGy (a), 4–15 mGy (b).

At lower doses (1 and 2 mGy), the FBP spectra of the CT Revolution showed a smaller amplitude but a stronger shift toward lower frequencies in comparison to those of the CT Discovery as displayed in Figs. 8 and 9 for kernel STD and SOFT respectively. Compared to the spectra of the CT Discovery obtained with BONE kernel, the ones of the CT Revolution present a completely different behavior with the presence of a second, low‐frequency, peak. (see Fig. 10).

At doses above 4 mGy, we observe a suppression of higher‐frequency component for the FBP reconstruction of the CT Revolution. This suppression is more evident passing from softer to sharper kernel (Figs. 8, 9, 10).

**Figure 11:**
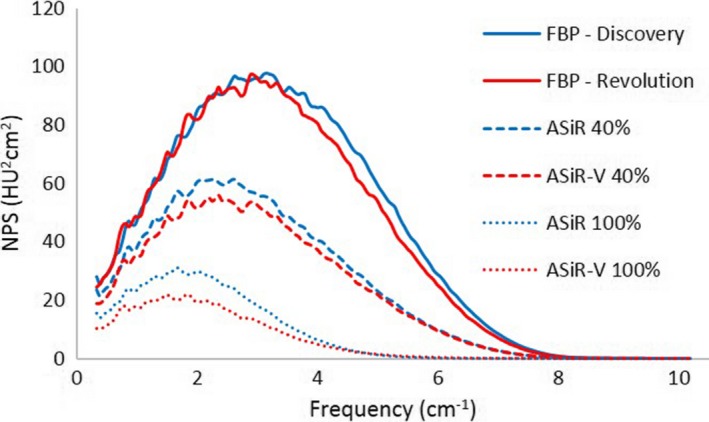
NPS with STD kernel for ASiR‐V and ASiR at different IR levels (CTDI_vol_ = 7 mGy).

**Figure 12:**
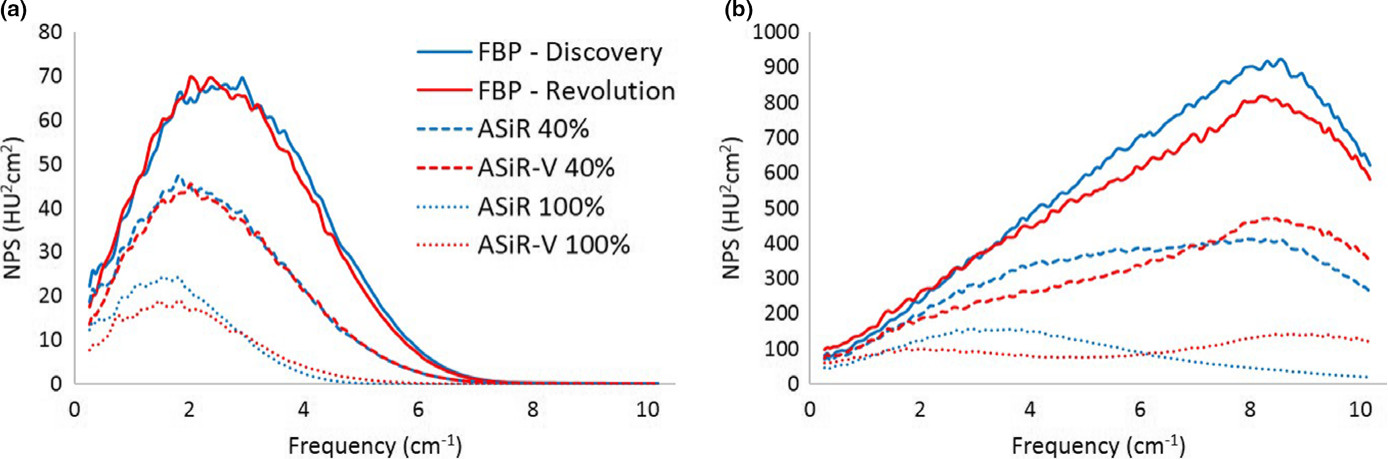
NPS with SOFT (a) and BONE (b) kernel for ASiR‐V and ASiR at different IR levels (CTDI_vol_ = 7 mGy).

Taking into account the intrinsic difference among the FBP spectra for the 2 scanners, at doses above 4 mGy, for the same level of the iterative, ASiR‐V shows basically the same NPS as ASiR, for kernel STD (Fig. 11) and SOFT [Fig. 12(a)]. The main difference between the NPS is observed with the BONE kernel [Fig. 12(b)].

At last, dependence on slice thickness was investigated.

NPS for ASiR‐V shows dependence on slice thickness: in particular, spectra show lower f_peak_ and f_mean_ for 2.5 mm, for kernel STD and SOFT. On the contrary, for BONE kernel the f_peak_ remains the same, while the f_mean_ decreases for slice thickness of 0.625 at the highest level of the IR.

ASiR, conversely, shows no dependence on slice thickness.

Values of f_peak_ and f_mean_ for the ASiR‐V for all kernels in the dose‐independent range (i.e., above 4 mGy), are shown in Table 3.

**Table 3:** ASiR‐V mean and peak frequency for CTDI_vol_ values above 4 mGy

IR level	Kernel STD				Kernel SOFT				Kernel BONE			
	f_peak_ (cm^−1^)		f_mean_ (cm^−1^)		f_peak_ (cm^−1^)		f_mean_ (cm^−1^)		f_peak_ (cm^−1^)		f_mean_ (cm^−1^)	
	0.625 mm	2.5 mm	0.625 mm	2.5 mm	0.625 mm	2.5 mm	0.625 mm	2.5 mm	0.625 mm	2.5 mm	0.625 mm	2.5 mm
0% (FBP)	2.8	2.8	3.2	3.2	2.4	2.4	2.8	2.8	8.4	8.4	6.4	6.4
4.0%	2.4	2.1	2.9	2.8	2.1	1.8	2.5	2.4	8.6	8.6	6.3	6.3
6.0%	2.1	1.8	2.7	2.5	1.9	1.6	2.4	2.2	8.8	8.8	6.1	6.2
100%	1.5	1.3	2.1	1.8	1.5	1.2	1.9	1.6	9.0	9.0	5.8	6.2

## 5 DISCUSSION

In this work, we assessed noise properties of ASiR‐V, a new GE iterative reconstruction algorithm, and compared them to those of FBP as well as ASiR.

At first, since the reconstruction with a given percentage of the IR algorithm means weighted blend of the FBP and the ASiR or ASiR‐V,^20^, ^21^ the behavior of the FBPs of the two CTs has to be taken into account.

For FBP at 1 and 2 mGy of the CT Revolution, in fact, the SD of HU as a function of CTDI_vol_ deviated from the power‐law σ ∝ D^−0.5^ and the NPS is shifted toward lower frequencies for STD and SOFT kernels, while for BONE kernel NPS presents a different shape. Above 4 mGy, instead, the relationship between SD and dose follows the well‐known power‐law and the NPS of the FBP was independent of dose, as expected.

This is consistent with the presence in the reconstruction process of a low‐pass filter that is noise dependent, until a certain threshold.

The particular trend for the FBP influences the behavior of the ASiR‐V, as noise reduction is lower for dose levels of 1 and 2 mGy, and NPS for the same IR level maintains the shift toward lower frequencies.

These properties are specific to the FBP reconstruction of the CT Revolution: since FBP reconstruction on the CT Discovery had the expected behavior (σ ∝ D^−0.5^ at all doses, and NPS of the FBP are not dose‐dependent at lower doses), all further comparisons between the algorithms were therefore carried out for doses above 4 mGy, where we observe a very small scanner‐specific behavior.

ASiR‐V reduces noise significantly, relative to FBP: the noise reduction was greater for higher levels of IR, with no dose dependence, for all kernels.

The impact of the ASiR‐V algorithm on noise was dependent on slice thickness: in particular, with the STD and SOFT kernels, we observed greater noise reduction at 2.5 mm ST than at 0.625, while for kernel BONE at the 2.5 mm, noise reduction is lower than at 0.625 mm.

Unlike ASiR‐V, noise reduction for ASiR showed no dependence on slice thickness.

Considering the comparison between ASiR‐V and ASiR, we observe greater noise reduction for the ASiR‐V with kernel STD and SOFT, at every IR level. On the other hand, if we consider kernel BONE, the impact of the ASiR‐V is lower than the one of the ASiR, especially at 2.5 mm ST.

The NPS behavior of ASiR‐V was similar to that of ASiR for the STD and SOFT kernels: at increasing level of the IR, the noise power spectra presents lower amplitude and a shift toward lower frequencies.

The greatest differences are observed at 100% IR level: with SOFT kernel, ASiR‐V presents more power at higher frequencies [Fig. 12(a)]. With BONE kernel the behavior is the opposite: more noise power at higher frequencies for the ASiR‐V, while ASiR has greater magnitude at lower frequencies [Fig. 12(b)].

As for the SD of HU units, the NPS for ASiR‐V shows dependence on slice thickness: spectra for kernel STD and SOFT calculated at 2.5 mm ST show shift toward lower frequencies that lead to lower values for f_peak_ and f_mean_.

Conversely, the dependence on slice thickness was not observed for ASiR.

The comparison of the NPS between the two algorithms in the range of small scanner‐dependent behavior (i.e., above 4 mGy) shows that the NPS with STD and SOFT kernels for the same IR level has the same shape, when calculated at 0.625 mm ST. The lower amplitude of the ASiR‐V NPS is due to the greater noise reduction in the ASiR‐V in comparison to ASiR, that lead to a smaller area under the curve.

With the BONE kernel we observe a substantial difference, with the NPS for ASiR‐V showing no shift of the high frequency peak and the formation of a second peak at lower doses.

All these results can be empirically explained considering the characteristics of the ASiR‐V with respect to the ASiR.

According to Fan,^15^ ASiR‐V is a successor to ASiR: it is based on the same architecture, with more advanced noise modeling and the addition of the physical modeling.

ASiR performance is not strongly influenced by the reconstruction kernel: in particular, the NPS is characterized by the shift toward lower frequencies also for BONE kernel, in the same fashion for STD and SOFT kernels.

In contrast, ASiR‐V more advanced noise modeling provides greater noise reduction compared to ASiR, and has a “kernel‐specific” effect that differs passing from low‐ to high‐frequency filters.

The improvement in modeling of noise statistics is reflected in the greater ASiR‐V noise reduction, for STD and SOFT kernels.

Noise reduction is also influenced by slice thickness: this is reasonably due to the regularization term, which encourages smoothness in the image and appears to be slice thickness dependent.

In addition, the fact that spectra for NPS at 2.5 mm present lower f_peak_ and f_mean_ is still due to the regularization term that add spatial correlation between neighboring voxels.

Considerations for kernel BONE are different and could be better explained starting from the performances of the ASiR.

For the ASiR‐V at a certain fixed IR level with kernel BONE, we observe greater noise at higher frequency, compared to ASiR.

Since the iteration process starts from the FBP image, it may be possible that ASiR‐V takes more into account the high‐frequency kernel, thus maintaining the high‐frequency peak, with a higher level of noise, typical for sharper filters. Conversely, ASiR has the same behavior (i.e., shifting toward lower frequencies), despite the FBP kernel. Thus, considering the peculiar shape of the NPS, the lower impact in noise reduction in ASiR‐V in comparison to ASiR, may not necessarily lead to worse image quality.

Lim et al.^22^ reported a significative increase in noise reduction in ASiR‐V in comparison to ASiR on phantom images, but IR levels were of 30%, 50% and 70% with slice thickness of 1.25 mm, so their results are not directly comparable with this study. Kwon et al.^16^ performed image reconstruction and noise evaluation in abdominal CT: slice thickness was 2.5 mm and ASiR‐V levels were of 30%, 50%, and 70%. They found a mean noise reduction in 34%, 46%, and 55% among different sites (liver, gluteal fat, and bladder), values slightly higher than our results (28% and 41% for ASiR‐V 40% and 60%, respectively) but noise was assessed on real patients and this may account for these differences.

Our results for noise reduction with ASiR are in good agreement with the values presented to date in the literature: Patino et al. reported a noise reduction in 39% for ASiR 60%, with measurements performed on an anthropomorphic abdomen phantom.^23^ Prakash et al. reported of a noise reduction in 21.5% using ASiR 30% on chest examinations.^24^ Mieville et al. reported a noise reduction in about 22% and 48% for ASiR 40% and ASiR 100% respectively.^25^

Results for NPS of ASiR with STD kernel are in good agreement with those of Mieville,^23^ while to our best knowledge there are no published paper for the NPS of the ASiR with kernels other than STD, and no papers at all for NPS of the ASiR‐V.

The main limitation of this study is that the ASiR‐V and ASiR are installed on two different scanners. We selected CTDI_vol_ to perform measurements under the same condition: discrepancy between scanner dose levels (i.e., CTDI values reported in console) is within 9% and better agreement is not possible since tube current works with steps of 5 mA.

If we consider CTDI_vol_ above 4 mGy (i.e., the range for which we have negligible scanner‐specific behavior), the greatest difference among the FBP noise levels is of 11.8% for kernel BONE at 15 mGy and 2.5 mm ST (33.09 vs 29.20 for CT Discovery and CT revolution respectively).

Anyway, since we calculated the noise reduction relative to FBP, results are reliable and describe appropriately the behavior of the IR algorithms.

Similar consideration has to be made for the NPS: since the two scanners are different, there are several aspects that may affect the NPS (bow‐tie filter design, x‐ray tube and detector characteristics, FBP reconstruction process). As shown in Figs. 8, 9, 10, FBP NPS for the CT Revolution present a slight suppression of high‐frequency components for STD and SOFT kernel (Figs. 8 and 9), while the suppression is more pronounced for BONE kernel (Fig. 10). This is true for doses above 4 mGy, while at lower doses, the noise‐dependent low‐pass filter of the FBP reconstruction for the CT Revolution, shifts dramatically the spectra.

Consequently, comprehensive comparison was carried out considering spectra starting from 4 mGy, for which we observe negligible scanner‐dependent behavior for the NPS of the FBP.

The second limitation of this study is that it is focused only on noise properties. Additional measurements should be performed to assess the signal properties of the ASiR‐V, in particular to study and evaluate spatial resolution. Since spatial resolution for the IR is contrast‐ and noise‐dependent due to the nonlinear regularization term, it is necessary to switch from the commonly used MTF metric to a “task‐based” metric that requires different noise and contrast levels with multiple acquisitions in order to obtain a reliable so‐called MTF_Task_, in particular when considering medium‐ and low‐contrast objects.^26^, ^27^

Lastly, physical characterization of IR algorithms performed in phantoms is only the starting point to allow full evaluation and optimization of clinical images. Comprehensive evaluation of IR in clinical routine requires observer models that predict detection accuracy, in order to allow also assessment of dose savings relative to FBP and among different reconstruction algorithms.

## 6 CONCLUSIONS

In conclusions, noise properties have been assessed for the new adaptive statistical iterative reconstruction, ASiR‐V, recently introduced on commercial CT systems.

The dependence of noise reduction and of the NPS have been investigated as a function of kernel, dose and slice thickness and comparison has been performed with ASiR.

ASiR‐V showed greater noise reduction than ASiR for STD and SOFT kernels, while keeping the same shape of the NPS and hence image texture. For the BONE kernel, ASiR‐V presents a completely different behavior, with less noise reduction and modified NPS.

In addition, noise properties of the ASiR‐V are dependent on reconstruction slice thickness.

The specific noise properties of ASiR‐V suggest the need for further measurements and efforts to establish new CT protocols to optimize clinical imaging.

## CONFLICT OF INTEREST

No conflict of interest.

## APPENDIX 1

### 1.1

**Table A1:** Noise for kernel STD at 0.625 mm expressed as mean SD

CTDI_vol_	FBP	ASiR 40%	ASiR 60%	ASiR 100%	FBP	ASiR‐V 40%	ASiR‐V 60%	ASiR‐V 100%
1 mGy	57.67	43.90	37.48	26.72	41.45	31.35	26.55	18.14
2 mGy	40.50	30.84	26.35	18.81	36.26	27.03	22.63	14.88
4 mGy	27.93	21.27	18.17	12.98	26.81	19.91	16.60	10.76
7 mGy	21.07	16.08	13.76	9.86	20.24	15.00	12.50	8.10
15 mGy	14.48	11.07	9.48	6.84	13.75	10.18	8.48	5.50

**Table A2:** Noise for kernel STD at 2.5 mm expressed as mean SD

CTDI_vol_	FBP	ASiR 40%	ASiR 60%	ASiR 100%	FBP	ASiR‐V 40%	ASiR‐V 60%	ASiR‐V 100%
1 mGy	37.19	28.34	24.21	17.30	25.78	18.94	15.71	10.30
2 mGy	26.15	19.96	17.07	12.23	22.11	15.98	13.06	8.19
4 mGy	18.04	13.78	11.79	8.46	16.25	11.66	9.49	5.84
7 mGy	13.59	10.39	8.90	6.42	12.23	8.76	7.13	4.40
15 mGy	9.33	7.16	6.15	4.47	8.29	5.95	4.86	3.01

**Table A3:** Noise for kernel SOFT at 0.625 mm expressed as mean SD

CTDI_vol_	FBP	ASiR 40%	ASiR 60%	ASiR 100%	FBP	ASiR‐V 40%	ASiR‐V 60%	ASiR‐V 100%
1 mGy	45.73	35.39	30.58	22.47	33.72	26.44	22.99	16.87
2 mGy	32.19	24.90	21.53	15.84	29.18	22.58	19.44	13.86
4 mGy	22.18	17.20	14.89	10.99	21.43	16.50	14.14	9.98
7 mGy	16.76	13.01	11.27	8.34	16.27	12.51	10.73	7.57
15 mGy	11.50	8.95	7.77	5.79	10.97	8.43	7.23	5.09

**Table A4:** Noise for kernel SOFT at 2.5 mm expressed as mean SD

CTDI_vol_	FBP	ASiR 40%	ASiR 60%	ASiR 100%	FBP	ASiR‐V 40%	ASiR‐V 60%	ASiR‐V 100%
1 mGy	29.54	22.89	19.80	14.59	21.06	15.89	13.45	9.31
2 mGy	20.81	16.14	13.97	10.31	17.88	13.28	11.11	7.43
4 mGy	14.33	11.14	9.65	7.16	12.99	9.59	7.99	5.27
7 mGy	10.82	8.42	7.31	5.44	9.84	7.27	6.06	4.00
15 mGy	7.44	5.82	5.06	3.81	6.63	4.90	4.09	2.72

**Table A5:** Noise for BONE at 0.625 mm expressed as mean SD

CTDI_vol_	FBP	ASiR 40%	ASiR 60%	ASiR 100%	FBP	ASiR‐V 40%	ASiR‐V 60%	ASiR‐V 100%
1 mGy	206.95	154.17	128.66	82.19	138.95	106.86	91.18	61.52
2 mGy	144.78	108.02	90.28	57.89	126.27	97.12	82.87	55.88
4 mGy	99.63	74.41	62.22	40.02	94.31	72.58	61.97	41.89
7 mGy	75.02	56.05	46.88	30.19	71.72	55.19	47.13	31.81
15 mGy	51.43	38.44	32.16	20.73	48.61	37.45	31.97	21.56

**Table A6:** Noise for kernel BONE at 2.5 mm expressed as mean SD

CTDI_vol_	FBP	ASiR 40%	ASiR 60%	ASiR 100%	FBP	ASiR‐V 40%	ASiR‐V 60%	ASiR‐V 100%
1 mGy	132.94	99.11	82.76	52.99	84.64	74.06	69.00	59.59
2 mGy	93.09	69.50	58.10	37.34	76.13	66.71	34.50	62.22
4 mGy	64.03	47.84	40.03	25.79	56.69	49.75	46.45	40.30
7 mGy	48.23	36.05	30.18	19.48	43.10	37.85	35.34	30.65
15 mGy	33.09	24.78	20.77	13.50	29.20	25.59	23.87	20.69
